# A retrospective study of the safety and efficacy of paclitaxel plus ramucirumab in patients with advanced or recurrent gastric cancer with ascites

**DOI:** 10.1186/s12885-018-4057-7

**Published:** 2018-01-31

**Authors:** Hiroshi Matsumoto, Akihito Kawazoe, Kaoru Shimada, Shota Fukuoka, Yasutoshi Kuboki, Hideaki Bando, Takashi Kojima, Atsushi Ohtsu, Takayuki Yoshino, Toshihiko Doi, Kohei Shitara

**Affiliations:** 1 Department of Gastroenterology and Gastrointestinal Oncology, National Cancer Center Hospital East, 6-5-1, Kashiwanoha, Kashiwa, Chiba 277-8577 Japan; 2 Department of Diagnostic Radiology, National Cancer Center Hospital East, 6-5-1, Kashiwanoha, Kashiwa, Chiba 277-8577 Japan

**Keywords:** Gastric cancer, Ascites, Progression-free survival, Paclitaxel, Ramucirumab

## Abstract

**Background:**

Ramucirumab has recently proved to be effective for advanced or recurrent gastric cancer (AGC). Ascites and peritoneal metastasis are among the most common complications of AGC. However, there are few data on the safety and efficacy of paclitaxel plus ramucirumab in patients with AGC with ascites. The purpose of this retrospective study was to evaluate the safety and efficacy of paclitaxel plus ramucirumab in patients with AGC with ascites.

**Methods:**

We retrospectively evaluated the safety and efficacy of paclitaxel plus ramucirumab in patients with AGC with ascites in comparison with patients without ascites in a single institution from June 2015 to May 2016. The median progression-free survival (PFS) and overall survival (OS) were calculated using the Kaplan-Meier method, and differences evaluated using the Log-lank test. The differences in baseline characteristics and response rates of each ascites group were calculated for homogeneity by chi-square tests and for trends by Fisher’s exact test.

**Results:**

Eighty-three patients were analyzed in this study. Ascites was detected in 40 patients, 26 patients (31%) had small to moderate ascites and 14 (17%) had massive ascites. The proportion of patients who started with a reduced dose of paclitaxel was higher for patients with massive ascites than others. The frequencies of any grade 3 or 4 hematological toxicity were 51% in patients without ascites, 77% in patients with small to moderate ascites, and 71% in patients with massive ascites. The frequencies of common ramucirumab-related adverse events were also not significantly different among ascites groups, however one patient had a tumor hemorrhage, and one patient had a gastrointestinal perforation. PFS and OS were shorter in patients with massive ascites than in patients with small or moderate ascites or patients without ascites.

**Conclusions:**

The use of paclitaxel and ramucirumab in patients with AGC with large amounts of ascites was tolerable with adequate dose modification. However, we should pay attention to the risks of ramucirumab-related toxicity in patients with bleeding tumors or intestinal stenosis.

## Background

Gastric cancer is the fifth most common cancer and the third leading cause of cancer death worldwide [1]. Currently, the first-line treatments for advanced or recurrent gastric cancer (AGC) are combination chemotherapy regimens consisting of fluoropyrimidines and platinum, with or without a third agents (trastuzumab if HER2 positive, or a taxane or anthracycline in some regions if HER2 negative) [2, 3], but the prognosis for patients with AGC remains poor, with median overall survival (OS) of 12–15 months in Asia (in Western Countries the median OS is less than 12 months in most studies) [4, 5].

Ascites and peritoneal metastasis are among the most common complications of AGC, resulting in several symptoms as well as critical complications, such as bowel obstruction, bile duct obstruction, and hydronephrosis. Several reports have suggested that the presence of ascites or peritoneal metastasis is associated with poor OS in AGC [6–9]. Therefore, it is important to develop effective treatment for ascites and peritoneal metastasis.

Ramucirumab is a human IgG1 monoclonal antibody specific for vascular endothelial growth factor receptor-2 (VEGFR-2), which has recently proved to be effective for AGC from the results of the REGARD and RAINBOW trials [10, 11]. In the RAINBOW trial, which compared paclitaxel plus placebo with paclitaxel plus ramucirumab, patients treated with paclitaxel plus ramucirumab showed significantly longer OS (median, 9.6 vs. 7.4 months), longer progression-free survival (PFS) (median, 4.4 vs. 2.9 months), and higher response rate (28% vs. 16%) than those treated with paclitaxel alone. Based on these results, paclitaxel plus ramucirumab became the standard second-line treatment for AGC. Because elevated levels of VEGFR2 ligands such as VEGF-A or VEGF-C in ascites have been reported to be associated with poor OS in AGC [12], the efficacy of ramucirumab for AGC with ascites is anticipated. However, detailed results of ramucirumab treatment in this patient population have not yet been reported, although one-third of patients in the RAINBOW trial had ascites. Moreover, because patients with large amounts of ascites were excluded from the RAINBOW study, the efficacy and feasibility of ramucirumab in this patient population are unclear. Therefore, we retrospectively evaluated the safety and efficacy of paclitaxel plus ramucirumab in patients with AGC with ascites.

## Methods

### Patients

This retrospective study was designed to evaluate the safety and efficacy of chemotherapy with paclitaxel plus ramucirumab in patients with AGC with ascites in comparison with patients without ascites. We reviewed the medical records of consecutive patients with AGC who had been treated with paclitaxel plus ramucirumab in a single institution from June 2015 to May 2016.

The eligibility criteria were the presence of histologically proven, inoperable AGC; Eastern Cooperative Oncology Group performance status (ECOG PS) of 0–2; adequate bone marrow, hepatic, and renal function; history of previous treatment with one or more regimens; and at least one treatment with paclitaxel plus ramucirumab. We excluded the patients with another disease which cause ascites: congestive heart failure, liver cirrhosis, nephrotic syndrome. Written informed consent for chemotherapy was obtained from each patient prior to the initiation of treatment. The study was performed under an institutional review board waiver in accordance with the Japanese ethical guidelines for epidemiologic research.

### Treatment plan

The patients received 8 mg/kg of ramucirumab intravenously on days 1 and 15, plus 80 mg/m^2^ of paclitaxel intravenously on days 1, 8, and 15 of a 28-day cycle. The patients received treatment until disease progression, unacceptable toxicity, or withdrawal of consent. The dose reduction of paclitaxel at starting the treatment was decided by each investigator depending on ECOG PS or toxicities from previous treatments (especially remaining sensory neuropathy due to oxaliplatin). Dose modification and interruption of treatment were performed by each investigator based on the criteria of reported clinical trials [11]. The patients could continue with ramucirumab or paclitaxel alone if they experienced severe toxicity with either agent.

### Evaluation of treatment and statistical analysis

The amount of ascites was defined as small (limited to the pelvic cavity or around the liver), moderate (neither small nor massive), or massive (continuous ascites from the surface of the liver to the pelvic cavity) by computed tomographic (CT) scans. These definitions are the same as those used in previous studies [13, 14]. The volume of ascites was estimated by the five-point method, as previously reported [14–16]. Briefly, the thickness of the ascites in centimeters was measured in three planes, the bilateral subphrenic space (A and B), the bilateral paracolic space (C and D), and the prebladder space (E). The average thickness, (A + B + C + D + E)/5, was then multiplied by the area of the standard abdominal cavity in the anterior projection, which was assumed to be 1000 cm^2^, to yield the volume of ascites as (A + B + C + D + E) × 200 (mL). We divided the patients into those without ascites, those with small or moderate ascites, and those with massive ascites.

Toxicities were graded according to the National Cancer Institute’s Common Terminology Criteria for Adverse Events, version 4.0. In patients with measurable lesions, tumor response was assessed according to the guidelines of the Response Evaluation Criteria in Solid Tumors (version 1.1), and the best overall response was recorded as the antitumor effect for that patient. The objective response rate in these patients was defined as the percentage of patients with a complete response (CR) or a partial response (PR). Changes in ascites were defined as follows: disappeared (disappearance of ascites), decreased (from moderate to small or from massive to moderate or small), and increased (from small to moderate or massive or from moderate to massive). The time to treatment failure (TTF) was determined from the date of initiation of chemotherapy to the date of the last administration of paclitaxel or ramucirumab. The actual dose intensity was defined as the total dose of drug delivered per unit of body surface area per unit of time (mg/m^2^/week). The relative dose intensity of paclitaxel was calculated as the ratio between the actual dose intensity and the scheduled dose intensity in patients who received at least one cycle of paclitaxel plus ramucirumab. PFS was measured from the date of initiation of chemotherapy to the date of disease progression or death from any cause. OS was estimated from the date of initiation of chemotherapy to the date of death or last follow-up visit. Median PFS and median OS were estimated by the Kaplan–Meier method. *P* values for testing differences in baseline characteristics and response rates of each ascites group were calculated for homogeneity by chi-square tests and for trends by Fisher’s exact test. PFS and OS were compared among ascites groups by the log-rank test. Hazard ratios (HRs) were calculated by the Cox proportional hazards model and presented as HRs and 95% confidence intervals (95% CIs). Statistical analyses were performed with IBM ® SPSS ® Statistics software (version 21). All tests were two-sided, and *P* < 0.05 was considered to indicate statistical significance.

## Results

### Patient characteristics

A total of 121 patients with AGC received ramucirumab-containing chemotherapy between June 2015 and May 2016. Of these patients, 83 received the chemotherapy with ramucirumab plus paclitaxel. They met the inclusion criteria and were analyzed in this study. The others were excluded due to different treatment regimens including irinotecan plus ramucirumab or ramucirumab monotherapy. Patient characteristics are shown in Table 1. Ascites was detected in 40 patients (49%); 14 patients (17%) had small ascites, 12 (14%) had moderate ascites, and 14 (17%) had massive ascites. The estimated median volumes of ascites according to this classification were 240 mL in small ascites (range, < 100–380 mL), 740 mL in moderate ascites (range, 320–1340 mL), and 2240 mL in massive ascites (range, 740–3340 mL). We could not estimate volumes of ascites in 10 patients with small ascites by this method because its distributions were out of described 5 points [14–16]. Four patients with massive ascites needed drainage of ascites before ramucirumab treatment after above-mentioned CT examination. The proportion of patients with ECOG PS 2 was higher among those with massive ascites than in other groups (*P* = 0.008) (Table 1). Fifty-nine patients (71%) had received one previous treatment with platinum-based and fluoropyrimidine-based chemotherapy regimens, and 24 patients (29%) had received previous treatment with two or more regimens. Peritoneal metastasis was diagnosed by laparotomy or laparoscopy in 16 patients. The other 33 patients were diagnosed by CT scans.

**Table 1:** Patients characteristics

		All (*N* = 83) (%)	No ascites (*N* = 43) (%)	Small to moderate ascites (*N* = 26) (%)	Massive ascites (*N* = 14) (%)
Age	Median (range)	67 (23–83)	68 (48–78)	64.5 (23–83)	67.5 (32–81)
Gender	Male*	60 (72)	37 (86)	15 (58)	8 (57)
ECOG PS	0*	48 (58)	28 (65)	18 (69)	2 (14)
	1	25 (30)	13 (30)	5 (19)	7 (50)
	2*	10 (12)	2 (5)	3 (12)	5 (36)
Histological Type	Diffuse	47 (57)	20 (46)	17 (65)	10 (71)
	Intestinal	36 (43)	23 (54)	9 (35)	4 (29)
Number of previous CTX	1	59 (71)	29 (67)	18 (69)	12 (86)
	≥2	24 (29)	14 (33)	8 (31)	2 (14)
Gastrectomy	Yes	31 (37)	18 (42)	7 (27)	6 (43)
Target lesion	Yes	45 (54)	28 (65)	12 (46)	5 (36)
Site of metastasis	Lymph node	56 (67)	34 (79)	15 (58)	7 (50)
	Liver	22 (27)	13 (30)	7 (27)	2 (14)
	Peritoneal	49 (59)	11 (26)	24 (92)	14 (100)
	Ovary*	10 (12)	1 (2)	5 (19)	4 (29)
Number of metastases	1–2*	70 (84)	39 (91)	19 (73)	12 (86)
	≥ 3	13 (16)	4 (9)	7 (27)	2 (14)

*CTX* chemotherapy

*p < 0.05

### Treatment results and toxicity

The median TTF among all patients was 3.7 months during the median follow-up period of 7.1 months. The proportion of patients who started with a reduced dose of paclitaxel was higher for patients with massive ascites (50%; 7 of 14 patients) than for patients with small to moderate ascites (19%; 5 of 26 patients; *P* = 0.043) or without ascites (19%; 8 of 43 patients; *P* = 0.021). The dose of paclitaxel was reduced during treatment in 49 patients (59%), with no significant differences between the ascites groups in the number of patients receiving reduced doses. In four patients, they discontinued ramucirumab due to bleeding or hemorrhagic events; three patients discontinued ramucirumab because of uncontrolled anemia due to oozing from primary lesions, and one patient discontinued ramucirumab because of a large amount of gastrointestinal hemorrhage due to an enlarged peritoneal metastasis that infiltrated into the small intestine. The median relative dose intensities of ramucirumab and paclitaxel were 87.9% (range, 14.3–100%) and 68.2% (range, 11.2–100%), respectively. Seventy-two patients discontinued treatment because of disease progression (*n* = 65; 90.3%), toxicity (*n* = 4; 5.6%), or other reasons (*n* = 3; 4.1%). The frequency of discontinuation due to toxicity was not significantly different among the ascites groups: 2 of 14 patients with massive ascites, 0 of 24 patients with small to moderate ascites, and 2 of 34 patients without ascites discontinued treatment.

The frequencies of any grade 3 or 4 hematological toxicity were 51% (22 of 43 patients) in patients without ascites, 77% (20 of 26 patients) in patients with small to moderate ascites, and 71% (10 of 14 patients) in patients with massive ascites (Table 2). The frequency of grade 3 or 4 febrile neutropenia was 4% (3 of 83 patients). The frequencies of any grade 3 or 4 nonhematological toxicity were 9% (4 of 43 patients) in patients without ascites, 15% (4 of 26 patients) in patients with small to moderate ascites, and 14% (2 of 14 patients) in patients in massive ascites. The adverse events of special interest that were potentially associated with ramucirumab are shown in Table 3. One patient without ascites had a grade 3 gastrointestinal hemorrhage resulting from an enlarged peritoneal metastasis that infiltrated into the small intestine 4 days after the last dose of ramucirumab. Another patient with small to moderate ascites had a gastrointestinal perforation due to disease progression and obstruction of the small intestine 10 days after the last ramucirumab treatment. Both patients received best supportive care and died 1 week and 2 weeks after these events.

**Table 2:** Adverse events

	All (*N* = 83) (%)		No ascites (*N* = 43) (%)		Small to moderate ascites (*N* = 26) (%)		Massive ascites (*N* = 14) (%)		*P* value^a^
	All (%)	Gr3-4 (%)	All (%)	Gr3-4 (%)	All (%)	Gr3-4 (%)	All (%)	Gr3-4 (%)	
Hematological toxicity									
Any	70 (84)	52 (63)	35 (81)	22 (51)	24 (92)	20 (77)	11 (79)	10 (71)	0.076
Neutropenia	61 (73)	40 (48)	30 (70)	19 (44)	21 (81)	19 (73)	10 (71)	8 (57)	0.064
Leukopenia	59 (71)	32 (39)	29 (67)	11 (26)	20 (77)	14 (54)	10 (71)	7 (50)	0.041
Anemia	27 (33)	8 (10)	15 (35)	4 (9)	9 (35)	3 (12)	3 (21)	1 (7)	0.49
Thrombocytopenia	8 (10)	3 (4)	4 (9)	3 (7)	3 (12)	0	1 (7)	0	0.72
Nonhematological toxicity									
Any	76 (92)	10 (12)	39 (91)	4 (9)	24 (92)	4 (15)	13 (93)	2 (14)	0.72
Fatigue	38 (46)	0	20 (47)	0	9 (35)	0	9 (64)	0	0.23^b^
Neuropathy	38 (46)	0	23 (53)	0	11 (42)	0	6 (43)	0	0.87^b^
Anorexia	37 (45)	6 (7)	17 (40)	2 (5)	12 (46)	3 (12)	8 (57)	1 (7)	0.56
Hypertention	24 (29)	2 (2)	11 (26)	0	10 (38)	1 (4)	3 (21)	1 (7)	0.27
Peripheral edema	23 (28)	0	11 (26)	0	7 (27)	0	5 (36)	0	0.74^b^
Proteinuria	9 (11)	0	4 (9)	0	5 (19)	0	0	0	0.12^b^
Diarrhea	8 (10)	0	6 (14)	0	1 (4)	0	1 (7)	0	0.36^b^
Epistaxis	8 (10)	0	5 (12)	0	1 (4)	0	2 (14)	0	0.62^b^
Febrile netropenia	3 (4)	3 (4)	3 (7)	3 (7)	0	0	0	0	0.26

^a^Comparison in grade 3 or 4

^b^Comparison in all grades

**Table 3:** Adverse events of special interest for ramucirumab

	All (*N* = 83) (%)		No ascites (*N* = 43) (%)		Small to moderate ascites (*N* = 26) (%)		Massive ascites (*N* = 14) (%)		*P* value*
	All (%)	≥ Gr3(%)	All (%)	≥ Gr3(%)	All (%)	≥ Gr3(%)	All (%)	≥ Gr3(%)	
Hypertension	24 (29)	2 (2)	11 (26)	0	10 (38)	1 (4)	3 (21)	1 (7)	0.27
Bleeding or haemorrhage	19 (23)	2 (2)	9 (21)	2 (5)	6 (23)	0	4 (29)	0	0.39
Proteinuria	9 (11)	0	4 (9)	0	5 (19)	0	0	0	0.16**
Liver injury or failure	6 (7)	0	2 (5)	0	2 (8)	0	2 (14)	0	0.48**
Gastrointestinal haemorrhage	5 (6)	2 (2)	3 (7)	2 (5)^a^	1 (4)	0	1 (7)	0	0.39
Gastrointestinal perforation	1 (1)	1 (1)	0	0	1 (4)	1 (4)	0	0	0.33
Infusion-related reaction	0	0	0	0	1 (4)	0	0	0	0.33**

*Comparison in grade 3 or more

**Comparison in all grades

^a^One patient died due to peritoneal metastasis which infiltrated to small intestine

### Efficacy

**Fig. 1:**
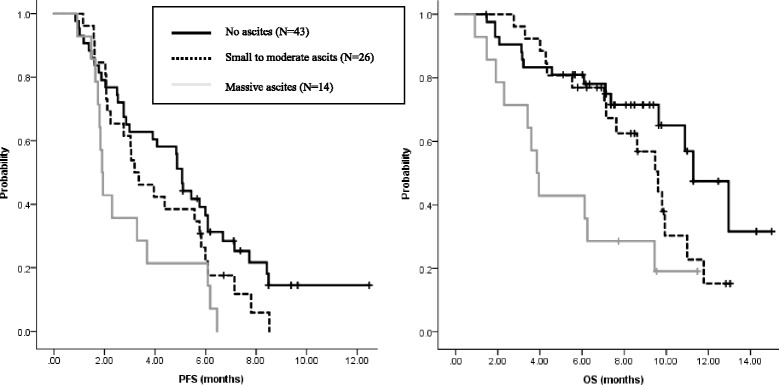
PFS and OS by ascites group. Progression-free survival by ascites group. Median PFS was shorter in patients with massive ascites (1.9 months; 95% CI, 1.7–2.1 months) than in patients with small or moderate ascites (3.2 months; 95% CI, 2.0–4.3 months; HR 0.57; 95% CI, 0.29–1.14; *P* = 0.11) or patients without ascites (5.1 months; 95% CI, 4.7–5.4 months; HR 0.65; 95% CI, 0.47–0.90; *P* = 0.01). Overall survival by ascites group. Median OS was shorter in patients with massive ascites (3.9 months; 95% CI, 3.2–4.5) than in patients with small or moderate ascites (9.6 months; 95% CI, 7.9–11.4 months; HR 0.41; 95% CI, 0.19–0.90; *P* = 0.026) or patients without ascites (11.3 months; 95% CI, 9.3–13.3 months; HR 0.54; 95% CI, 0.36–0.81; *P* = 0.003)

Of the 45 patients with measurable lesions, 14 achieved a PR with an overall response rate of 31.8%. Of the patients with ascites (*n* = 40), disappearance of ascites was observed in 3 patients (8%), and a decrease of ascites was observed in 11 patients (28%). Among 4 patients requiring drainage of ascites, 2 patients became free from drainage. The response rates in terms of measurable lesions or ascites were similar among the ascites groups (Tables 4 and 5). Seventy patients had experienced disease progression at the time of analysis, with a median PFS of 4.0 months (95% CI, 2.5–5.4 months). Forty-two patients (50.1%) were dead, with a median OS of 9.6 months (95% CI, 9.2–10.1 months). The median PFS was shorter in patients with massive ascites (1.9 months; 95% CI, 1.7–2.1 months) than in patients with small or moderate ascites (3.2 months; 95% CI, 2.0–4.3 months; HR 0.57; 95% CI, 0.29–1.14; *P* = 0.11) or patients without ascites (5.1 months; 95% CI, 4.7–5.4 months; HR 0.65; 95% CI, 0.47–0.90; *P* = 0.01) (Fig. 1). The median OS was also shorter in patients with massive ascites (3.9 months; 95% CI, 3.2–4.5 months) than in patients with small or moderate ascites (9.6 months; 95% CI, 7.9–11.4 months; HR 0.41; 95% CI, 0.19–0.90; *P* = 0.026) or patients without ascites (11.3 months; 95% CI, 9.3–13.3 months; HR 0.54; 95% CI, 0.36–0.81; *P* = 0.003) (Fig. 1). Forty patients (56%) received post-discontinuation therapy, most commonly with irinotecan-based chemotherapy (*n* = 26). Fewer patients with massive ascites received post-discontinuation chemotherapy (29%; 4 of 14 patients) than patients with small or moderate ascites (67%; 16 of 24 patients) or without ascites (59%; 20 of 34 patients), although the difference was not statistically significant (*P* = 0.065).

**Table 4:** Response in measurable lesions

Groups	N	CR	PR	SD	PD	ORR (%)	DCR (%)	*P* value*
All patients	45	0	14	22	9	31.8	77.3	0.54
No ascites	28	0	11	14	3	39.2	89.2	
Small to moderate ascites	12	0	2	6	4	16.7	66.7	
Massive ascites	5	0	1	2	2	20.0	60.0	

*CR* complete response, *PR* partial response, *SD* stable disease, *PD* progressive disease, *ORR* objective response rate, *DCR* disease control rate (CR + PR + SD)

*Comparison of ORR between 3 groups

**Table 5:** Response in ascites

Groups	N	Disappeared (N) (%)	Decreased (N) (%)	No change (N) (%)	Increased (N) (%)	NE (N) (%)	*P* value*
All patients with ascites	40	3 (7.5)	11 (27.5)	12 (30)	12 (30)	2 (5)	0.88
Small to moderate ascites	26	3 (12)	6 (23)	9 (35)	8 (31)	0	
Massive ascites	14	0	5 (36)	3 (21)	4 (29)	2 (14)	

Disappeard: disappearance of ascites

Decreased: from moderate to small, from massive to moderate or small

Increased: from small to moderate or massive, from moderate to massive

*Comparison of response between 2 groups

## Discussion

We retrospectively evaluated the safety and efficacy of chemotherapy with ramucirumab plus paclitaxel for AGC patients with ascites. Our primary interest was the feasibility of this treatment for patients with large amounts of ascites. In this study, the frequencies of grade 3 or more adverse events, except for leukopenia, were not significantly different across ascites groups, and the frequency of treatment discontinuation because of adverse events was also not significantly different across ascites groups. However, a significantly higher fraction of patients with massive ascites started with a reduced dose of paclitaxel based on poor ECOG PS status; therefore, these results should be interpreted cautiously. The frequencies of common ramucirumab-related adverse events, such as hypertension, proteinuria, and bleeding, were also not significantly different among ascites groups and were comparable to those of the RAINBOW and REGARD trials. Importantly, one patient had a tumor hemorrhage and one patient had a gastrointestinal perforation, although a causal relation between these events and ramucirumab treatment was not definite because these events occurred on disease progression in both patients. These events are rare but well-known adverse events related to the inhibition of the VEGF pathway, which had been reported in the RAINBOW study. Although the precise risks of severe bleeding and gastrointestinal perforation related to the use of ramucirumab for AGC have not yet been analyzed in detail, our patients had some characteristics, such as tumor infiltration to the small intestine and stenosis due to peritoneal metastasis. Therefore, the indication of ramucirumab treatment should be carefully considered for patients who are at risk of bleeding tumors or gastrointestinal stenosis due to peritoneal metastasis.

The response rates in terms of measurable lesions were similar among the ascites groups. Disappearance or decreased amount of ascites was observed in 38% patients, which suggested the efficacy of this treatment for this patient population. Efficacy against ascites was previously reported for paclitaxel monotherapy [15]; therefore, the exact impact of ramucirumab on this patient population should be explored by further analysis in cohorts with larger sample sizes. PFS and OS in patients with massive ascites were significantly shorter than those in patients with smaller amounts of ascites. More effective treatments are needed to improve the poor prognosis of this patient population.

There are several limitations to this study. First, this was a retrospective analysis in a single institution with a small sample size. In this study, 29% of patients had received two or more previous treatment regimens. This study included ECOG PS 2 patients, who were excluded from the RAINBOW trial. These differences are possible reasons for the shorter PFS and OS in this study than those in the RAINBOW trial. Second, several patients started with a reduced dose of paclitaxel based on the physician’s judgment. Therefore, the frequencies of adverse events might be lower than those associated with the standard dose of paclitaxel. However, the dose of ramucirumab was not reduced; therefore, there might be little influence of dose of paclitaxel on the analysis of ramucirumab-related toxicities.

## Conclusion

The use of paclitaxel and ramucirumab in patients with AGC with large amounts of ascites was tolerable with adequate dose modification. However, we should pay attention to the risks of ramucirumab-related toxicity in patients with bleeding tumors or intestinal stenosis.

## Abbreviations

AGC: Advanced or recurrent gastric cancer
CIs: Confidence intervals
CR: Complete response
CT: Computed tomographic
ECOG PS: Eastern Cooperative Oncology Group performance status
HRs: Hazard ratios
OS: Overall survival
PFS: Progression-free survival
PR: Partial response
TTF: Time to treatment failure
VEGFR-2: Vascular endothelial growth factor receptor-2
